# Supplementary material for: Optical control of resonances in temporally symmetry-broken metasurfaces
Source: Nature. 2025 Aug 6;644(8078):896–902. doi: 10.1038/s41586-025-09363-7 (PMC12390841; doi:10.1038/s41586-025-09363-7)
Supplement: Supplementary file 1 — Supplementary Notes 1–10. [file 41586_2025_9363_MOESM1_ESM.pdf]

nature portfolio

https://doi.org/10.1038/s41586-025-09363-7

Supplementary information

Optical control of resonances in temporally symmetry-broken metasurfaces

In the format provided by the authors and unedited

Supplementary Information: Optical Control of Resonances in Temporally Symmetry-Broken Metasurfaces

Andreas Aigner[1,+], Thomas Possmayer[1,+], Thomas Weber[1], Alexander A. Antonov[1]
Leonardo de S. Menezes[1,2], Stefan A. Maier[3,4,], and Andreas Tittl[1,*]*

1) Chair in Hybrid Nanosystems, Faculty of Physics, Ludwig-Maximilians-University Munich, 80539 Munich, Germany.
2) Departamento de Física, Universidade Federal de Pernambuco, 50670-901 Recife-PE, Brazil.
3) School of Physics and Astronomy, Monash University, Clayton, Victoria 3800, Australia.
4) Department of Physics, Imperial College London, London SW7 2AZ, United Kingdom.

[+] authors contributed equally

[*] stefan.maier@monash.edu, andreas.tittl@physik.uni-muenchen.de

Supplementary Note 1: TCMT model

The following section is based on the work of Fan *et al.* on temporal coupled-mode theory for the Fano resonance in optical resonators[1]. We use a single resonator coupled to two ports, allowing transmission and reflection. The resonance is excited through port 1, with the incoming wave represented as $\boldsymbol{s}_+ = (s_{1+}, 0)^{\mathrm{T}}$, and the output waves in port 1 (reflected wave) and port 2 (transmitted wave) are represented by $\boldsymbol{s}_- = (s_{1-}, s_{2-})^{\mathrm{T}}$, with s_{1+}, s_{1-}, and s_{2-} as the incoming, reflected, and transmitted wave's amplitude, respectively.

The resonant mode's time-dependent amplitude, denoted as $a(t)$, temporally evolves according to

$$\frac{\mathrm{d}a(t)}{\mathrm{d}t} = (i\omega_0 - \gamma_{\mathrm{tot}})\, a(t) + \boldsymbol{\kappa}^{\mathrm{T}} \boldsymbol{s}_+$$

where ω_0 is the resonance frequency, $\gamma_{\mathrm{tot}} = \gamma_{\mathrm{rad}} + \gamma_{\mathrm{int}}$ is the total decay rate as the sum of radiative loss γ_{rad} and intrinsic loss γ_{int}. The outgoing waves $\boldsymbol{s}_-$ are related to the incoming waves and the resonator amplitude via

$$\boldsymbol{s}_- = C\boldsymbol{s}_+ + a(t)\boldsymbol{\kappa}.$$

Here, $\boldsymbol{\kappa} = \left(\sqrt{\gamma_{\mathrm{rad}}}, \sqrt{\gamma_{\mathrm{rad}}}\right)^{\mathrm{T}}$ is the radiative damping rate describing the coupling between the ports and the mode while C represents the nonresonant port-to-port coupling with

$$C = e^{i\varphi} \cdot \begin{pmatrix} r_0 & it_0 \\ it_0 & r_0 \end{pmatrix}.$$

r_0 and t_0 are the background reflection and transmission, respectively, with $r_0^2 + t_0^2 = 1$, while φ represents a global phase.

Using the above formulas and assuming a time-harmonic mode amplitude, $\frac{\mathrm{d}a(t)}{\mathrm{d}t} = i\omega a(t)$, the equation for the resonance's time-dependent amplitude can be written as

$$a(t) = \frac{\boldsymbol{\kappa}^{\mathrm{T}} \boldsymbol{s}_+}{(i\omega - i\omega_0 - \gamma_{\mathrm{tot}})}.$$

Assuming unitary transmission for off-resonance wavelengths and using $s_{2-} = \sqrt{\gamma_{\mathrm{rad}}}\,a(t)$, we can substitute $a(t)$ and eventually calculate the transmission coefficient $t(\omega) = \frac{s_{2-}}{s_{1+}}$ as

$$t(\omega) = 1 - \frac{\gamma_{\mathrm{rad}}}{i(\omega - \omega_0) + \gamma_{\mathrm{tot}}} = 1 - \frac{\gamma_{\mathrm{rad}}}{i(\omega - \omega_0) + \gamma_{\mathrm{rad}} + \gamma_{\mathrm{int}}}.$$

Supplementary Note 2: Behavior of optical modes as a function of asymmetry

The defining property of symmetry-protected BICs is that the radiative Q-factor scales with the asymmetry parameter α via $Q_{rad} \sim \alpha^{-2}$. As the structure in our RSP-BICs is geometrically asymmetric, we have to redefine α as the deviation from the symmetry-protected state: In the case of the width sweep, it is simply the difference between w_1 and w_2, where $\alpha = 0$ corresponds to $w_1 = w_2 = 95$ nm, and α increases as w_1 increases. In the case of the length sweep, we define the symmetric length as the RSP-BIC length, $l_{2,\mathrm{RSP-BIC}} = 216$ nm, and α as $l_2 - l_{2,\mathrm{RSP-BIC}}$.

Figure S1 revisits the simulated Q_{rad} (**Figure 2e)**, however now with the respective asymmetry factors. To reveal the SP-BIC's typical $Q_{rad} \sim \alpha^{-2}$ dependence, both axes are plotted on a logarithmic scale, which results in a linear relationship, with the slope representing the exponent of the power law. For both the width and length sweep, the fitted Q_{rad} values (black dots) lie on a straight line for smaller asymmetries. The gray curve is a fit excluding high asymmetries (gray dots) for the width sweep, where the exponential trend breaks down, suggesting that α is no longer a suitable asymmetry factor in this range. The fit for the width sweep (**Figure S1a**) yields an exponent of -1.95, close to the theoretical value of -2. For the length sweep (**S1b**), the fitted exponent is -2.22 for negative α, and -1.96 for positive ones. This demonstrates that Q_{rad} behaves similarly around the SP-BIC and RSP-BIC conditions.

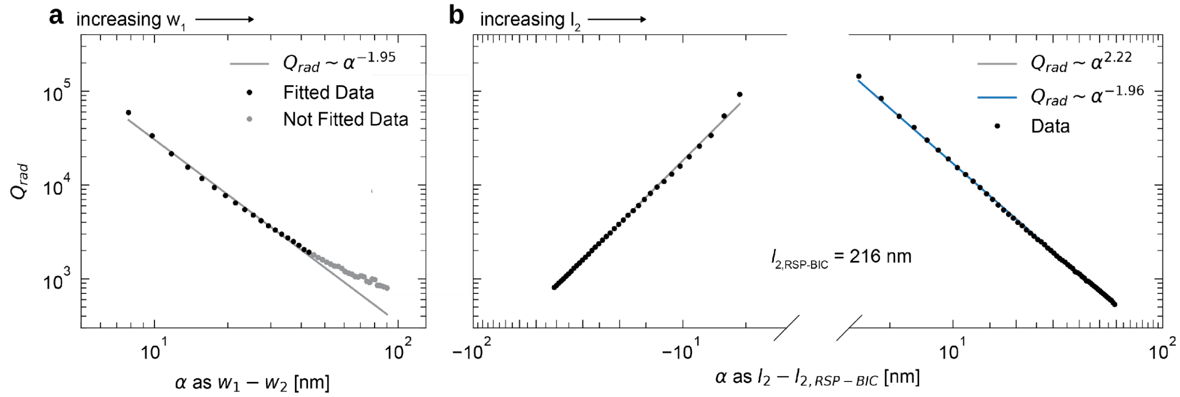

Figure S1: Relation of radiative loss and asymmetry. In **(a)**, the width of the first resonator is varied. **(b)** Corresponding plots for the l_2 sweep from **Figure 2e**. In (b) the data is separated into two sets: $\alpha < 0$ and $\alpha > 0$. The region around $\alpha = 0$ is cropped for clarity.

Additionally, both the RSP-BIC and the SP-BIC exhibit similar field profiles, shown in **Figure S2**, where electric field intensities and directions are plotted in the SP-BIC, RSP-BIC and two quasi-BIC conditions. All four cases display a similar antiparallel dipolar mode profile.

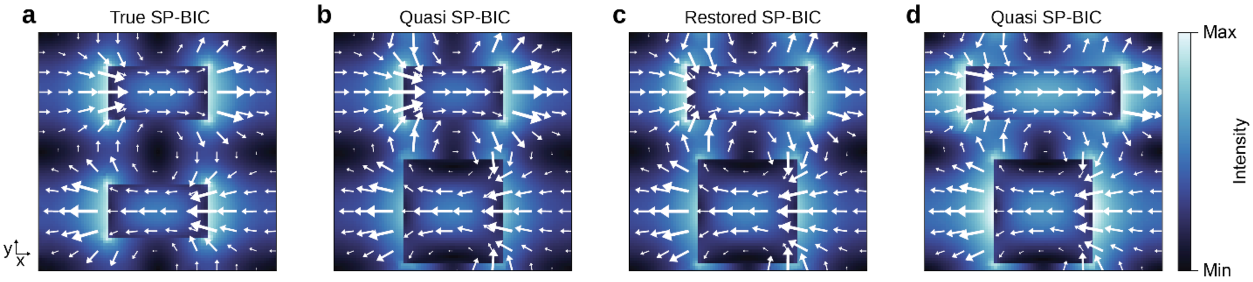

Figure S2: Electric field profile of the SP-BIC mode. Electric field intensity profiles ($|E|^2$) within the x-y plane at $z = 57.5$ nm (center height) of the unit cell for different resonance conditions at their specific resonance wavelengths. The field intensity is color-coded from minimum to maximum within each individual cut. The direction of the electric field is represented by white arrows, with the arrow size indicating the field strength. **(a)** The true SP-BIC condition, structurally and optically symmetric, featuring two antiparallel dipole modes, one in each rod. **(b)** The quasi-BIC condition, reached by increasing w_1, breaking the structural symmetry. **(c)** The RSP-BIC condition, obtained by subsequently increasing l_2, restoring the optical symmetry. **(d)** The quasi-BIC condition after further increasing l_2.

Supplementary Note 3: Resonant State Expansion

To describe how a change in the permittivity $\Delta\varepsilon$ of the rods during optical pumping affects the RSP-BIC, we implement Resonant State Expansion (RSE) theory, assuming a metasurface with constant refractive index of silicon ($n_0 = 3.7$) embedded in vacuum to eliminate any parasitic open channels associated with diffraction.

First, we analyze the eigenmodes of the metasurface using the Electromagnetic Waves Frequency Domain module in COMSOL Multiphysics. **Figure S3a** shows a colormap of the quasi-BIC Q-factor as a function of the width of the first rod w_1 and the length of the second rod l_2. All other geometrical parameters remain the same as in the main text: a square unit cell with a period of 420 nm, $l_1 = 175$ nm, $w_2 = 95$ nm, and a height of the rods of 115 nm. In this 2D w_1-l_2 parameter space, the RSP-BIC condition with a Q-factor $> 10^7$ is represented by a continuous line. The parameters for the RSP-BIC ($w_1 = 185$ nm and $l_2 = 210.5$ nm) closely match those of the original structure on a sapphire substrate embedded in SiO_2 ($w_1 = 185$ and $l_2 = 216$ nm). The new RSP-BIC has a resonance wavelength of 637 nm and the complex eigenfrequency ω_2. **Figure S3b** shows that increasing either parameter redshifts the resonance wavelengths.

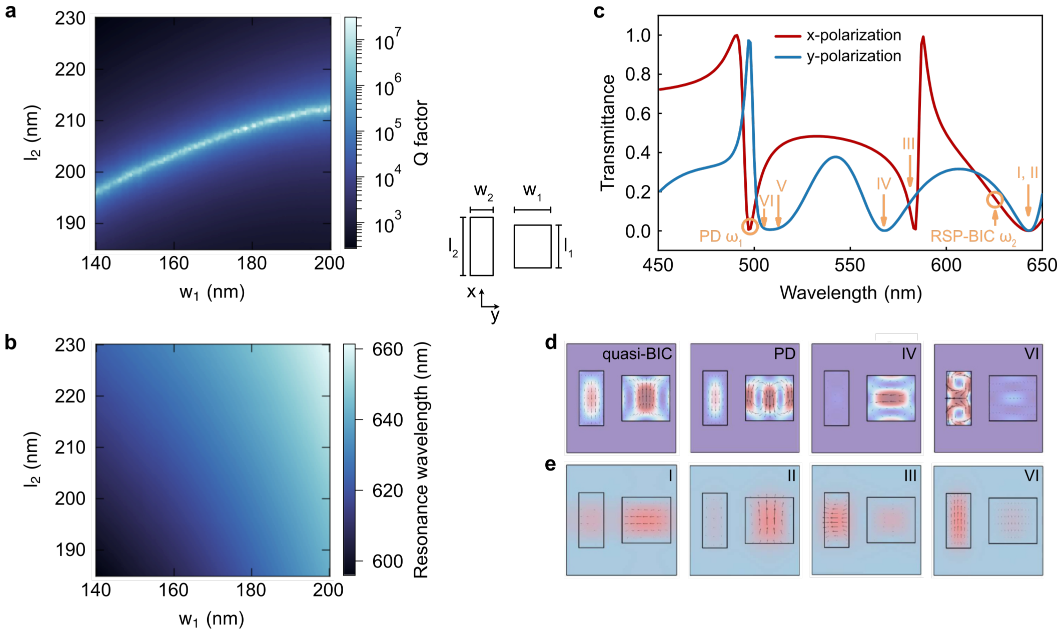

Figure S3: Analysis of eigenstates of the metasurface without perturbation ($\Delta\varepsilon = 0$). (a) Q-factor and **(b)** resonance position of the quasi-BIC as functions of the geometric parameters w_1 and l_2. The logarithmic colormap of the Q-factor shows the condition for the RSP-BIC as a continuous line in the w_1-l_2 parameter space. **(c)** Transmittance spectra of the metasurface with $l_2 = 210.5$ nm and $w_1 = 185$ nm for x and y-linearly polarized light. Labels indicate the positions of the "electric" and "magnetic" eigenstates, whose electric and magnetic field distributions over the center cut of the nanorods are shown in **(d)** and **(e)**, respectively.

RSE is a powerful tool to describe the hybridization of a finite number of metasurface eigenstates by introducing a perturbation. By changing $\Delta\varepsilon$ in the first rod, we preserve all three mirror symmetry planes along the main axes. Therefore, an antiparallel dipole quasi-BIC, whose electric field is predominantly along the x direction and has the components $E_{x,y}(x,y,z) = E_{x,y}(x,y,-z)$,

can only couple with an eigenstate of the same parity. The closest candidate is a parallel dipole (PD) resonance at 495 nm with the complex eigenfrequency ω_1. In **Figure S3c** we plot the metasurface's x- and y-polarized transmittance spectra and show corresponding eigenmodes at the marked spectral positions. We classify these eigenmodes by their current distribution over a mid-plane cross-section of the nanorods in the xy-plane as either "electric" (see **Figure S3d**) or "magnetic" (see **Figure S3e**). Due to removing the substrate and coverage, the quasi-BIC has no transparent background and lies near the "magnetic" resonance I. However, such a change does not affect our subsequent eigenstates analysis.

We define super-vectors $\mathbf{F}_1$ and $\mathbf{F}_2$, $\mathbf{F}_n(\mathbf{r}) = \{\mathbf{E}_n(\mathbf{r}), i\mathbf{H}_n(\mathbf{r})\}$ for the PD resonance and antiparallel dipole quasi-BIC, respectively. To apply RSE to metasurfaces as open optical resonators, the states with eigenfrequencies ω_n must be correctly normalized. We perform the normalization described in:[2]

$$1 = ||\mathbf{F}_n||^2 = \int_V [\varepsilon(\mathbf{r})\mathbf{E}_n \cdot \mathbf{E}_n - \mathbf{H}_n \cdot \mathbf{H}_n]\, dV + \frac{ic}{\omega_n}\int_{\partial V} [\mathbf{E}_n \times (\mathbf{r}\cdot\nabla)\mathbf{H}_n + \mathbf{H}_n \times (\mathbf{r}\cdot\nabla)\mathbf{E}_n] \cdot d\mathbf{S}, \quad (1)$$

where V is the volume of a unit cell with surface boundaries ∂V. We also consider nonmagnetic materials with the permeability $\mu = 1$.

We introduce a variation $\Delta\varepsilon$ in the first rod as an environmental perturbation and, as described by Gorkunov *et al.*[2], use first-order perturbation theory based on the RSE. The hybridized states $\tilde{\mathbf{F}}_n$ are expressed as a linear superposition of the states $\mathbf{F}_1$ and $\mathbf{F}_2$ of the unperturbed system with $\Delta\varepsilon = 0$:

$$\tilde{\mathbf{F}} = \sum_{m=1}^{2} a_m \mathbf{F}_m\,, \quad (2)$$

where we have truncated the RSE matrix equation to the two relevant states and keep coefficients a_n as a solution of a system of equations valid in the first order:

$$\omega_n a_n = \tilde{\omega} \sum_{m=1}^{2} (\delta_{nm} + V_{nm}) a_m, \quad (3)$$

In Eq. (3) δ_{nm} is the Kronecker delta and elements of the perturbation matrix read as:

$$V_{nm} = \int_V \Delta\varepsilon(\mathbf{r})\mathbf{E}_n(\mathbf{r}) \cdot \mathbf{E}_m(\mathbf{r}) dV\,, \quad (4)$$

where the integration is performed by a volume of the first rod V with environmental perturbation $\Delta\varepsilon$.

Assuming constant $\Delta\varepsilon$ we introduce new parameters to the perturbation matrix:

$$v_1 = \int_V \mathbf{E}_1(\mathbf{r}) \cdot \mathbf{E}_1(\mathbf{r}) dV, \quad v_2 = \int_V \mathbf{E}_2(\mathbf{r}) \cdot \mathbf{E}_2(\mathbf{r}) dV\,, \quad u = \int_V \mathbf{E}_1(\mathbf{r}) \cdot \mathbf{E}_2(\mathbf{r}) dV\,. \quad (5)$$

By solving Eq. (3), we find a doublet of hybrid eigenfrequencies:

$$\omega_{\pm} = \frac{1}{2(1-\tilde{u}^2)}\left[\widetilde{\omega}_1 + \widetilde{\omega}_2 \pm \sqrt{(\widetilde{\omega}_1 - \widetilde{\omega}_2)^2 + 4\tilde{u}^2\widetilde{\omega}_1\widetilde{\omega}_2}\right], \quad (6)$$

where the frequency parameters $\widetilde{\omega}_{1,2} = \omega_{1,2}\left[1 + v_{1,2}\Delta\varepsilon\right]^{-1}$ describe the spectral shift produced by the perturbation and the parameter $\tilde{u} = u\Delta\varepsilon/\sqrt{(1+v_1\Delta\varepsilon)(1+v_2\Delta\varepsilon)}$ determines the mixing of the original eigenstates.

We empirically find that the absolute values of the parameters of the perturbation matrix (5) of normalized states $\mathbf{F}_1$ and $\mathbf{F}_2$ do not exceed 0.03. Moreover, all the parameters (5) are always multiplied by an environmental permittivity perturbation $\Delta\varepsilon$ in accordance with (4). Therefore, we expand the expression from Eq. (6) as well as $\tilde{u}$ and $\widetilde{\omega}_{1,2}$, neglect all cubic terms in $\Delta\varepsilon$ and obtain the final expression for the hybrid eigenfrequencies:

$$\omega_{PD} \approx \omega_1\left[1 - v_1\Delta\varepsilon - \frac{u^2\omega_1(\Delta\varepsilon)^2}{\omega_1 - \omega_2}\right] \quad (7)$$

$$\omega_{qBIC} \approx \omega_2\left[1 - v_2\Delta\varepsilon - \frac{u^2\omega_2(\Delta\varepsilon)^2}{\omega_1 - \omega_2}\right] \quad (8)$$

We now evaluate how introducing $\Delta\varepsilon$ in the first rod hybridizes the eigenmodes. According to our findings from **Figure 3f**, dissipation losses γ_{int}, compared to the radiative losses γ_{rad}, return to their original values much faster after optical pumping. Therefore, we consider only the real part of $\Delta\varepsilon$, given by $\Delta\varepsilon = (n_0 - \Delta n)^2 - n_0^2 \approx -2n_0\Delta n$.

In **Figure S4a and S4b** we plot the resonance position and the imaginary part (γ_{rad}) of the hybrid eigenfrequencies as functions of the perturbation Δn, obtained through numerical simulations in COMSOL Multiphysics and the RSE theory from Eqs. (7) and (8), for both RSP-BIC and PD resonances. As shown, the RSE theory accurately reproduces the full-scale numerical modeling for both the real and imaginary parts of the eigenfrequencies.

Introducing Δn shifts the resonance position for both resonances in the shorter wavelength region: the RSP-BIC and PD resonance are shifted by 8 nm and 15 nm, respectively. The radiative part of the PD resonance depends linearly on the perturbation, while γ_{rad} of the RSP-BIC exhibits, at first sight, an almost quadratic dependence on the perturbation, which is typical of regular BIC behavior.[3] In **Figure S4c** we plot the Q-factor as a function of Δn on a logarithmic scale and reveal that the function depends on the perturbation in a more peculiar manner than inverse quadratic law $Q \propto 1/(\Delta\varepsilon)^2$.

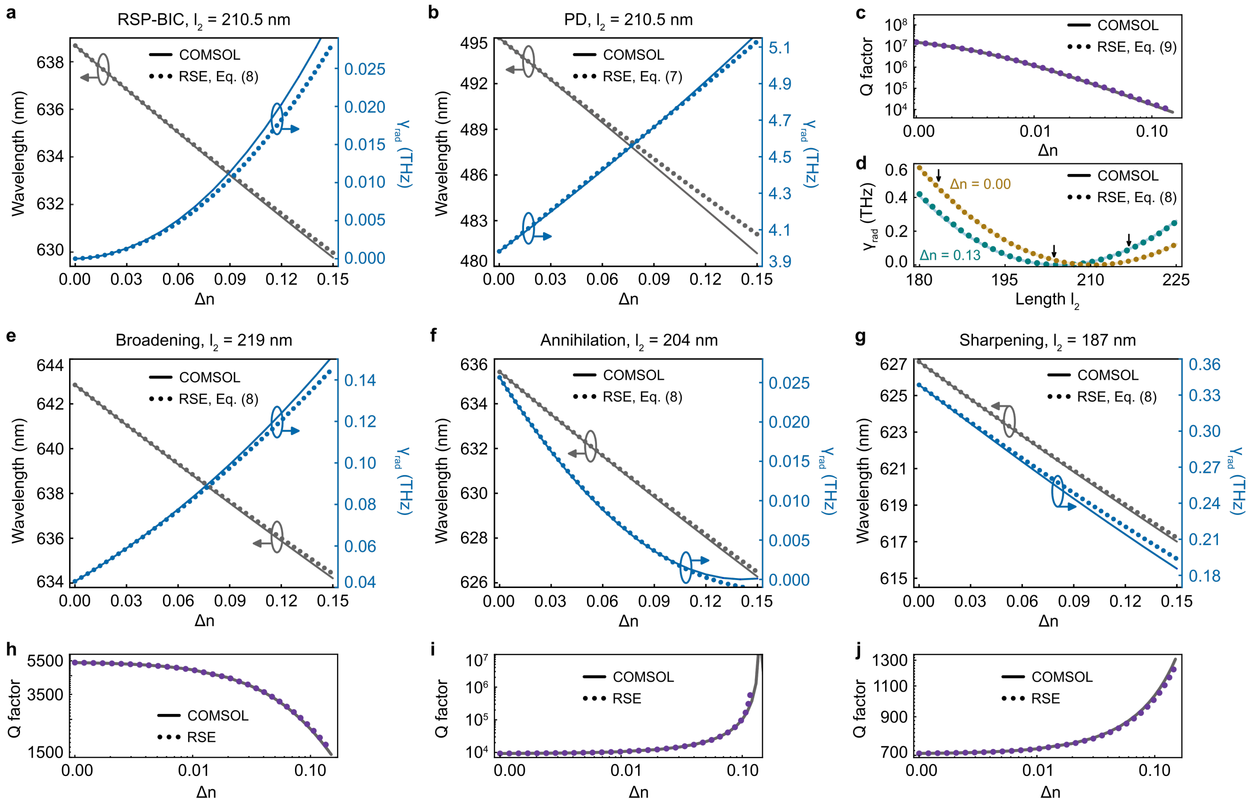

Figure S4: Analysis of hybrid eigenstates of the metasurface with perturbation Δn. Resonance position and the imaginary part (γ_{rad}) of the eigenfrequencies as functions of the perturbation Δn of the **(a)** RSP-BIC and **(b)** PD modes, obtained via COMSOL Multiphysics (solid lines) and RSE theory according to Eqs. (7) and (8) (dashed lines). **(c)** Q-factor of the RSP-BIC as a function of Δn obtained from both approaches. **(d)** γ_{rad} as a function of the second rod length l_2 for both the stationary case ($\Delta n = 0$) and perturbed case ($\Delta n = 0.13$). Arrow marks indicate the l_2 values used for the RSP-BIC **(e)** broadening, **(f)** annihilation and **(g)** sharpening processes. The final row **(h)-(j)** shows the Q-factor as a function of Δn for each of these processes. The width of the first rod is fixed everywhere as $w_1 = 185$ nm.

To explain this, we analyze the hybrid eigenfrequency of the quasi-BIC from Eq. (8). The electric fields of a true BIC are supposed to be real, which leads to a purely real parameter v_2. However, we analyze here the RSP-BIC with a large but still finite Q-factor $Q_0 = \omega_2'/(2\gamma_2')$ (where the complex eigenfrequency of RSP-BIC is $\omega_2 = \omega_2' + i\gamma_2'$), so the fields of the eigenmode still have a small imaginary part. This results in a complex $v_2 = v_2' + iv_2''$, $v_2' \sim 10^{-2}$ and $v_2'' \sim 10^{-6}$. One may fairly point out that v_2'' can be neglected. However, as we will show below, this component contributes to the peculiar behavior of the Q-factor and causes its deviation from the typical inverse square law. In addition to v_2, the eigenfrequency of the PD resonance ω_1 and the overlap integral u between the states also have the imaginary parts. For the sake of brevity, we introduce $\alpha = u^2\omega_2(\omega_1 - \omega_2)^{-1} = \alpha' + i\alpha''$. Then the Q-factor of the quasi-BIC reads as:

$$Q_{qBIC} \approx \frac{1}{\frac{1}{Q_0} - 2v_2''\Delta\varepsilon - 2\alpha''(\Delta\varepsilon)^2}. \tag{9}$$

Now one can see, that:

(i) In the case of $\Delta\varepsilon = 0$, the Q-factor of the quasi-BIC returns to the finite Q-factor Q_0 of the RSP-BIC;

(ii) Due to the nonzero value of $v_2'' \sim 10^{-6}$, the linear term in $\Delta\varepsilon$ plays an important role at small perturbation values, and the Q-factor function deviates from the typical inverse square law;

(iii) At relatively large $\Delta\varepsilon$, the main contribution comes from a quadratic term with α'', which includes a mixing parameter u between the PD resonance and the quasi-BIC;

(iv) In the case of a perfect BIC with $v_2'' = 0$ and $Q_0 \to \infty$, the Q-factor indeed exhibits the typical dependence on perturbation $Q \propto 1/(\Delta\varepsilon)^2$.

Finally, we plot the Q-factor of the RSP-BIC with $l_2 = 210.5$ nm as a function of Δn according to the analytical expression (9) and demonstrate a close match between the RSE theory and full-scale simulations.

Next, using Eq. (8), we plot $\gamma_{\rm rad}$ of the quasi-BIC as a function of l_2 for stationary ($\Delta n = 0$) and perturbated ($\Delta n = 0.13$) problems (**Figure S4d)**. According to the main text, we investigate three other different scenarios (in addition to the case of resonance creation, shown in **Figure S4a-S4c**): (i) broadening the resonance width (increase in $\gamma_{\rm rad}$ with initially nonzero radiative losses), (ii) resonance annihilation (decrease of $\gamma_{\rm rad}$ to zero), and (iii) resonance sharpening (decrease of $\gamma_{\rm rad}$ to a nonzero value). For all three cases, we fix l_2 at (i) 219 nm, (ii) 204 nm and (iii) 187 nm, respectively (see arrow marks in **Figure S4d**). As before, we use the RSE theory and compare it with COMSOL results. In all three cases, the RSE theory closely matches the full-scale simulations. It also predicts that the perturbation $\Delta n = 0.13$ increases $\gamma_{\rm rad}$ up to 0.12 THz for the "broadening" case and decreases $\gamma_{\rm rad}$ down to 0 THz or 0.20 THz for the "annihilation" and "sharpening" cases, respectively, which closely matches the experimental data fitting, shown in **Figure 5** of the main text.

One may notice that the RSE theory predicts negative $\gamma_{\rm rad}$ values for the "annihilation" case when $\Delta n \geq 0.14$, which has no physical meaning and demonstrates a slight overreach beyond the theory's limits of applicability. Additionally, in **Figure S4h-j** we plot the Q-factor dependencies on the perturbation Δn in logarithmic scale.

We separately consider the case where the change in $\Delta\varepsilon$ is due to the dissipation losses only. We introduce an environmental perturbation as $\Delta\varepsilon = (n_0 + i\Delta k)^2 - n_0^2$, and obtain the expected result: Δk doesn't significantly shift the resonance position of the RSP-BIC ($l_2 = 210.5$ nm) and, therefore, does not introduce a radiative part $\gamma_{\rm rad}$. At the same time, the imaginary part of the eigenfrequency $\gamma_0 = \gamma_{\rm rad} + \gamma_{\rm int} \approx \gamma_{\rm int}$ consists mostly on the intrinsic component and depends linearly on Δk (see **Figure S5**).

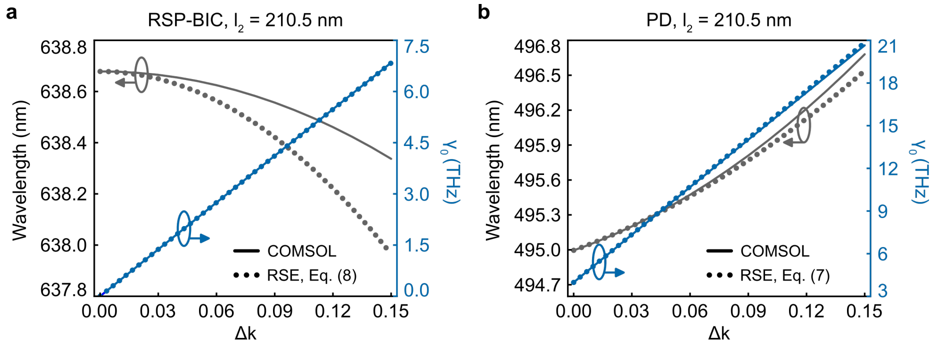

Figure S5: Analysis of hybrid eigenstates of the metasurface with dissipation losses Δk as an environmental perturbation. Resonance position and the imaginary part of the eigenfrequencies ($\gamma_0 = \gamma_{\text{rad}} + \gamma_{\text{int}} \approx \gamma_{\text{int}}$) of the **(a)** RSP-BIC and **(b)** PD modes, obtained via COMSOL Multiphysics (solid lines) and RSE theory (dashed lines), as functions of the extinction coefficient variation Δk.

As a conclusion of this part, we have demonstrated:

(i) The RSE theory can qualitatively reproduce the change in eigenfrequency due to environmental perturbation in one of the nanorods. It also shows that the introduction of a perturbation mixes the antiparallel dipole quasi-BIC and PD resonance.

(ii) The RSP-BIC Q-factor depends on the perturbation in accordance with Eq. (9), which deviates from the typical inverse quadratic law.

(iii) The agreement between the experimental data for γ_{rad} and the values predicted by the RSE theory for broadening, annihilation and sharpening cases allows us to evaluate the change in the nanorod's refractive index due to pumping as $\Delta n \sim 0.1$.

(iv) The introduction of Δk as an environmental perturbation results solely in a linear dispersion of the modes' intrinsic losses.

Supplementary Note 4: Multipole decomposition

To study the multipolar composition of the present photonic modes in the metasurface, we calculate the scattered power of one unit cell of the metasurface

$$P_{\text{scat}} = \frac{1}{2}\sqrt{\frac{\varepsilon_0 \varepsilon}{\mu_0}} \int |\mathbf{E}_{\text{scat}}|^2 d\Omega$$

where we decompose the scattered electric fields into several multipolar moments[4]

$$\mathbf{E}_{\text{scat}}(\mathbf{r}) = \frac{k_0^2 e^{ikr}}{4\pi\epsilon_0 r}\left([\mathbf{n} \times [\mathbf{p} \times \mathbf{n}]] + \frac{1}{v}[\mathbf{m} \times \mathbf{n}] + \frac{ik}{2}\left[\mathbf{n} \times \left[\mathbf{n} \times \widehat{\mathrm{Q}}_e \mathbf{n}\right]\right] + \frac{ik}{2v}\left[\mathbf{n} \times \widehat{\mathrm{Q}}_m \mathbf{n}\right] + \frac{k^2}{6}\left[\mathbf{n} \times \left[\mathbf{n} \times \widehat{0}_e \mathbf{nn}\right]\right] \right)$$

with **p** being the electric dipole moment (ED), **m** the magnetic dipole moment (MD), $Q_{e/m}$ the electric/magnetic quadrupole moments (EQ/MQ) and O_e the electric octupole moment (EO). The multipole moments are spatial integrals over the current density

$$\mathbf{J} = -i\omega\varepsilon_0(\varepsilon_r - \varepsilon)\mathbf{E}$$

where **E** is the electric field inside the resonator with ε_r denoting the relative permittivity of the resonator material and ε the relative permittivity of the environment.

Figure S6 shows the calculated contribution of the individual modes to the total scattered power from Rod 1 of the RSP-BIC in x- and y-polarization. Normalizing the mode's contributions to the total scattered power demonstrates that the single resonance in x-polarization is dominated by the magnetic dipole MD, whereas the two resonances in y-polarization are mostly governed by the ED component. However, the left resonance at around 400 THz exhibits a dip in ED power and a peak in MD power, to which we assign the term MD-like, and ED-like to the right resonance at around 405 THz.

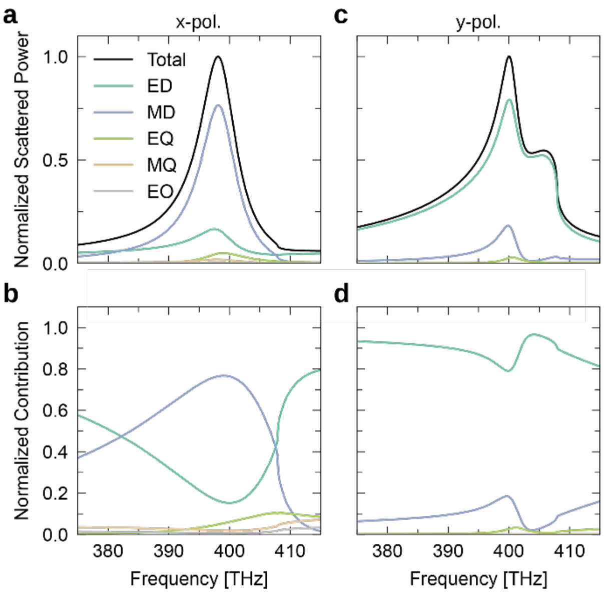

Figure S6: Mode decomposition. Normalized scattered power of individual multipolar components for Rod 1 with $l_2 = 216\,\mathrm{nm}$ for excitations in x-polarization **(a)** and y-polarization **(c)**. **(b)**, **(d)** Relative contributions $P_{\mathrm{multipole}}/P_{\mathrm{total}}$ to the total scattered power.

Supplementary Note 5: Off-Resonant Pumping

To modify the asymmetry of the structure, the pump laser should be resonant with the ED-like Mie mode 1; detuning it will reduce the amount of field-enhancement in rod 1, and therefore lead to a smaller change in the Q-factor of the quasi-BIC, as well as a smaller spectral shift. This is investigated experimentally in **Figure S7**, where we observe the maximum effect of our pump beam to be around 730 nm, while pump-induced changes in Q-factor or modulation amplitude are negligible below 710 nm.

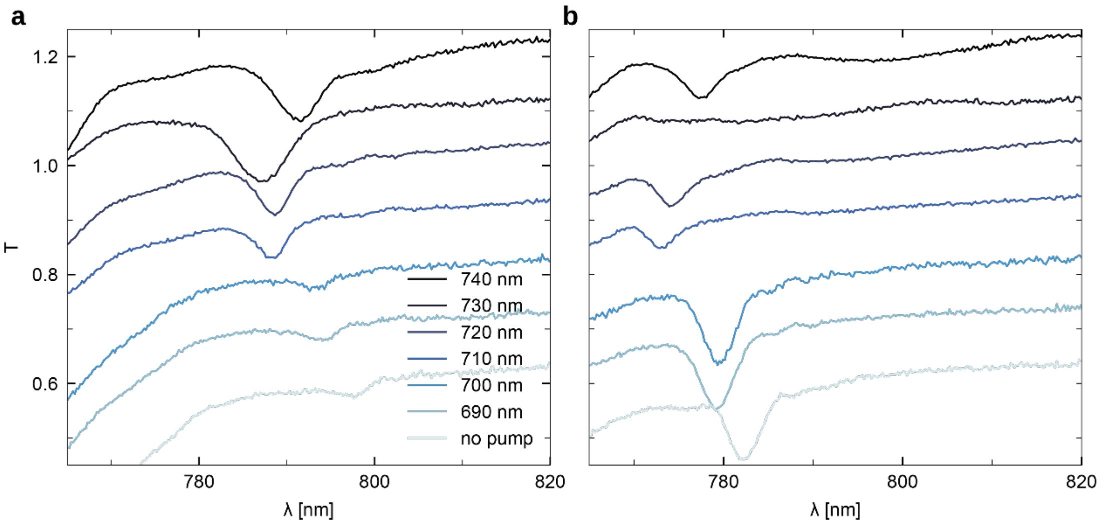

Figure S7: Wavelength-dependent pumping. Transmission spectra at different pump wavelengths at a fixed time delay of $t = 1$ ps and a fluence of 100 µJ/cm^2. **(a)** Resonance broadening position ($l_2 > l_{2,\mathrm{RSP-BIC}}$) and **(b)** resonance sharpening position ($l_2 < l_{2,\mathrm{RSP-BIC}}$). Consecutive spectra have been offset by 0.1 for clarity.

Supplementary Note 6: TCMT fitting of temporal evolution.

Extracting radiative and non-radiative losses from the measured transient absorption traces requires fitting the spectra at each time delay with the TCMT theory discussed in **Supplementary Note 1**. **Figures S8** and **S9** show the good convergence of the fit to the experimental data, which results in low relative errors (**Figure S10**).

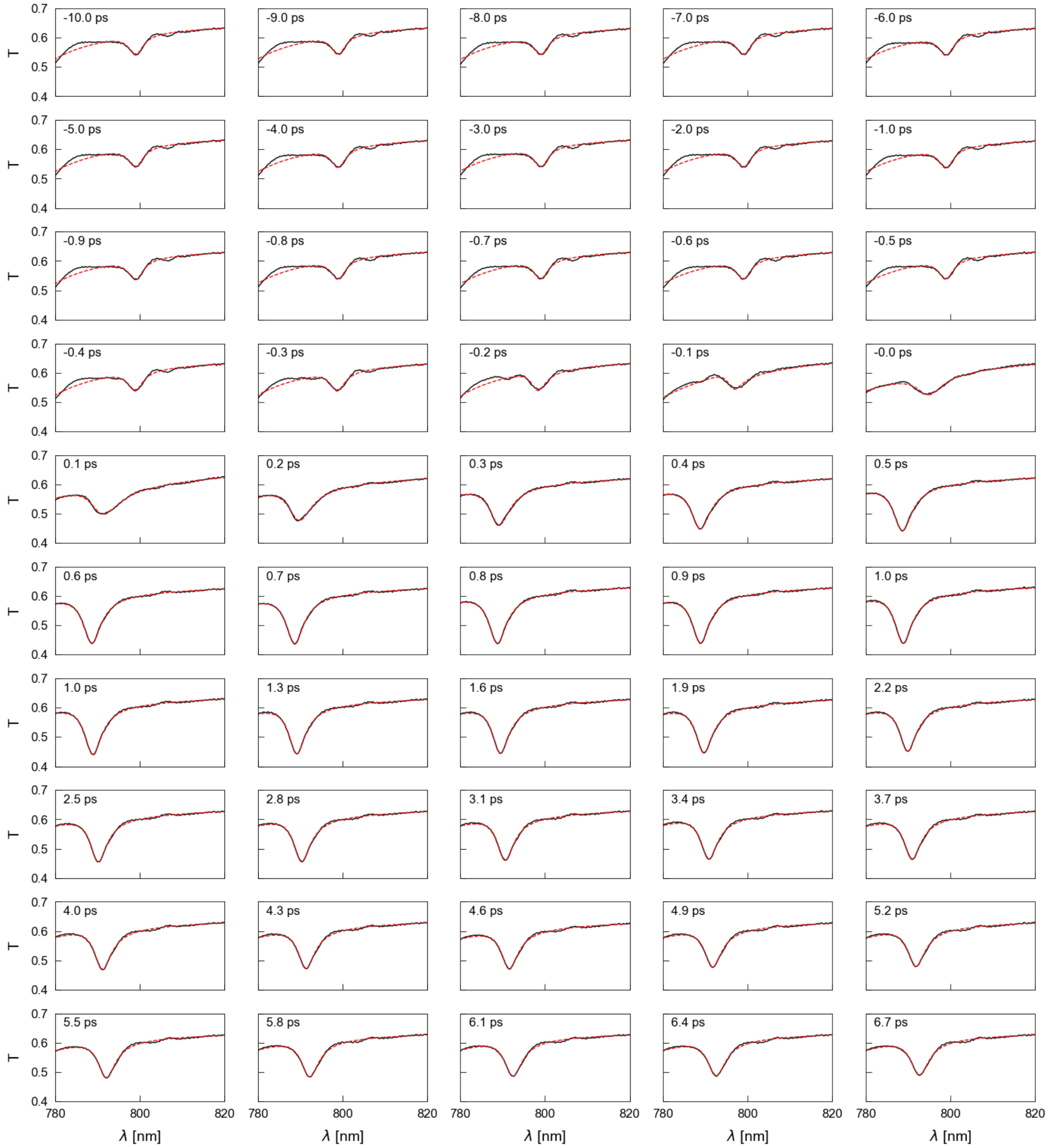

Figure S8: Temporal evolution of spectra and TCMT fits 1. Experimental spectra (black), and TCMT fits (red) for the time evolution from -10 ps to 6.7 ps, for $l_2 = 236$ nm. The data set is part of the time trace shown in **Figure 3e** and **Figure 5b**.

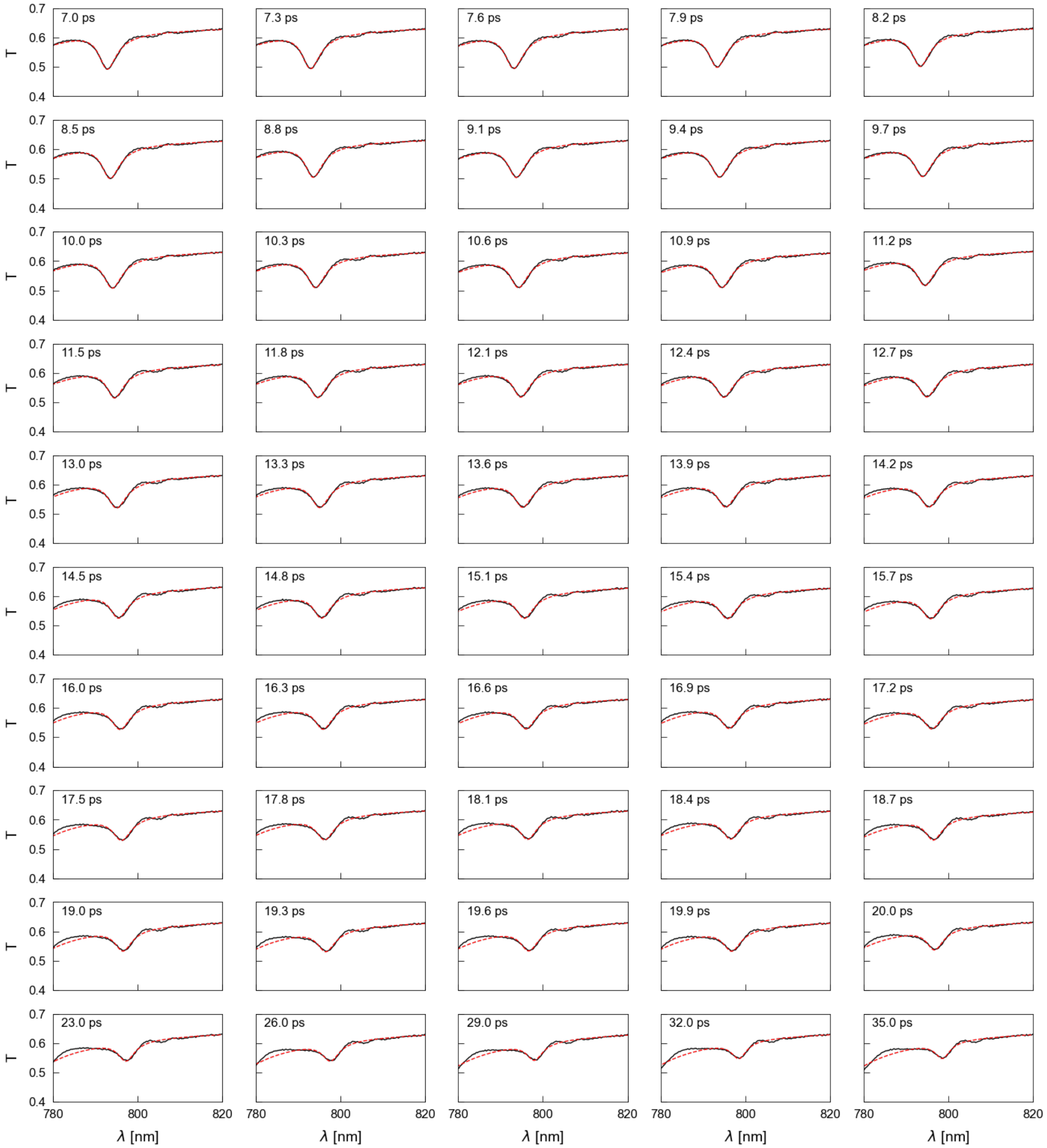

Figure S9: Temporal evolution of spectra and TCMT fits 2. Experimental spectra (black), and TCMT fits (red) for the time evolution from 7 ps to 35 ps, for $l_2 = 236$ nm. The data set is part of the time trace shown in **Figure 3e** and **Figure 5b**.

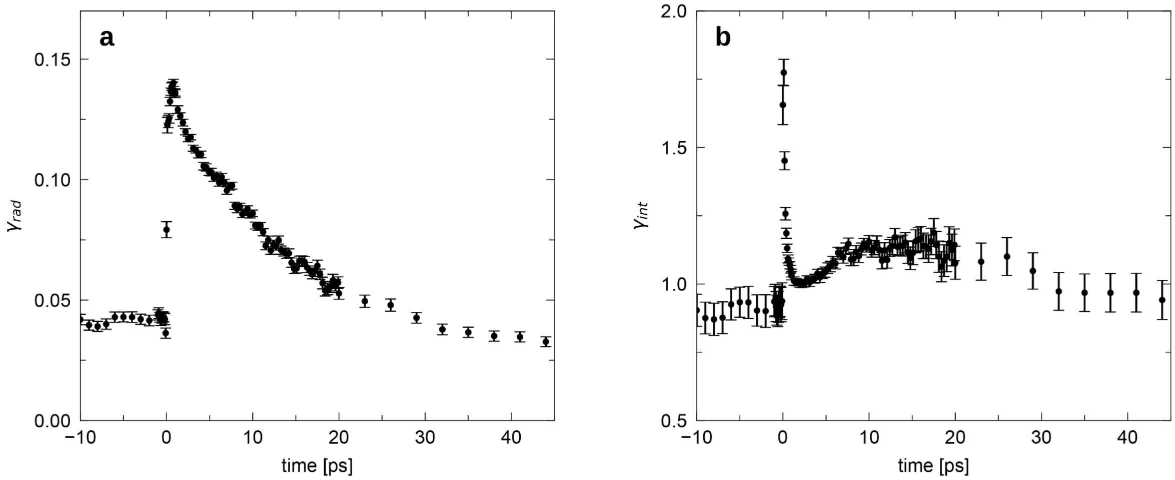

Figure S10: Standard errors of fitted data. Standard errors obtained for (a) γ_{rad}, and (b) γ_{int} using the TCMT model and the non-linear least-squares minimization package lmfit. The mean standard error for γ_{rad} is 0.0019 (2.4%), while the mean error for γ_{int} is 0.039 (3.6%).

Supplementary Note 7: Temporal resonance evolution

Pump-induced changes in the radiative loss rates of the metasurfaces affect both width and depth of the resonance, and are accompanied by changes in non-radiative losses. To visualize the overall performance of the pump to change the mode, we therefore first take a closer look at the combined Q-factor of the resonance in the broadening and sharpening condition (**Figure S11**). In the former case, the Q-factor decreases from an initial value of 400 down to 200 upon temporal overlap of the pump and probe pulses. It then stabilizes around 300 before gradually increasing back to its initial value of 400. In the latter case, the Q-factor rises from an initial 110 to 250, then gradually converges back to the initial value.

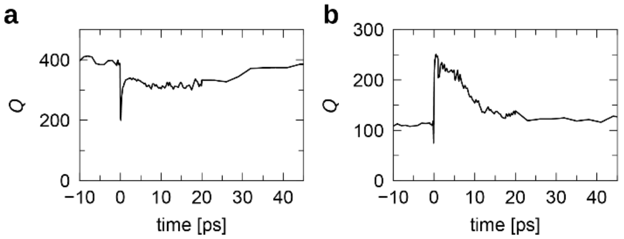

Figure S11: Total Q-factors during mode broadening and sharpening. (a) Total Q-factor obtained using the TCMT model with variable γ_{rad} and γ_{int} for the transmittance time trace shown in **Figure 5b** with $l_2 = 236$ nm. **(b)** Equivalent representation to (a), but for $l_2 = 186$ nm and the time trace of **Figure 5e**.

As discussed in **Supplementary Note 3**, changes in the refractive index in rod 1 give rise to a change in radiative properties of the mode. A close examination of the observed behavior allows to draw conclusions on the physical processes involved in the modulation and observed decay, but requires differentiating between radiative and non-radiative losses.

First, analogous to **Figure 3f**, we now take a look at the absolute values of the two loss rates for an exemplary geometry that enables an increase in modulation depth upon pumping. As seen in **Figure S12**, γ_{rad} first increases in a pulse-length limited timeframe, and decays with a time constant <10 ps, with some deviations from pure exponential decay as expected due to the nonlinear relationship between Δn and γ_{rad} in the broadening case (see **Supplementary Note 3**).

Since the modulation remains unchanged for lower repetition rates, thermal effects can be excluded. Furthermore, we see no pulse-length limited peak, rendering the Kerr-effect insignificant. This is expected from our structure needing a refractive index change of 0.18 to achieve the shown shift (see **Supplementary Note 8**), which is beyond what is achievable at our intensities with Kerr of lattice distortion effects like electrostriction, but can be realized with laser-induced carrier concentrations in the order of 10^{20} cm^{-3},[5] which is below what is observed in laser ablation experiments in silicon.[6] Lattice distortion effects might however contribute to minor longer-lasting changes in the radiative losses.

Dissipation losses γ_{int}, on the other hand, are significantly larger than γ_{rad} at the start of the experiment, which shows that the structure operates in the undercoupled regime. Consequently,

changes in absorption due to the increased carrier concentration in the conduction band after pumping only gives rise to a minor relative change in γ_{int}. However, we observe a large spike in γ_{int}, that is limited by the pulse duration. This is attributed to three effects: First, as the probe pulse around 800 nm is still in the low-absorption range of crystalline silicon, nondegenerate two-photon absorption can temporarily increase nonradiative losses while the pump pulse is also present in the sample. Second, the rapid shift of the resonance on timeframes shorter than our probe pulse causes an effective mode broadening through both the probe pulse being too long to resolve this process, as well as now non-resonant optical radiation scattering out of the resonator. Third, electron-electron scattering processes happen on timescales of similar length as our pump pulse, and can lead to ultrafast changes in absorptive properties.

Remarkably, γ_{int} first decreases to a value around 14% above its baseline after this initial spike, and continues increasing for the first 10 ps after the pump arrives. We attribute this slow rise to the cooling of the hot electron distribution, which happens in the timescale of a few picoseconds[7] and can impact absorptive properties. Only the subsequent decay is then analogous to the carrier decay in the refractive index modulation.

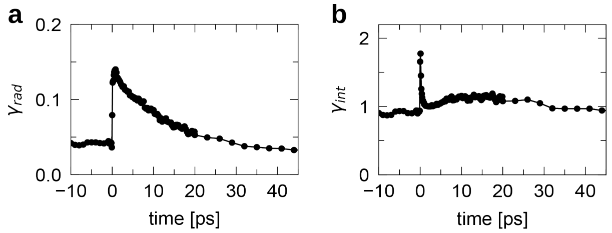

Figure S12: Radiative and intrinsic loss comparison. For $l_2 = 236$ nm, the transmittance time trace is fitted using the TCMT model with variable γ_{rad} and γ_{int}. (a) displays γ_{rad}, while (b) shows γ_{int}.

This extraction further allows gaining insights in the evolution of the field enhancement (FE) within the structure by estimating $FE \propto \sqrt{\gamma_{\text{rad}}}/(\gamma_{\text{rad}} + \gamma_{\text{int}})$ (see **Figure S13** for the FE from **Figure S12**). As this metric is a combination of radiative and non-radiative losses, we see effects from both loss rates: The initial spike in γ_{int} causes an ultrafast drop in the field enhancement, while the later increase and decay are mostly governed by the evolution of γ_{rad} due to the small relative change in γ_{int}.

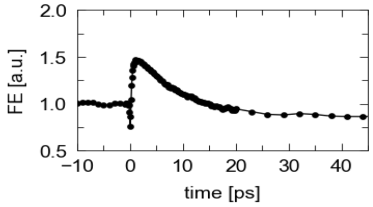

Figure S13: Estimated FE change. For $l_2 = 236$ nm, the previously extracted γ_{rad} and γ_{int} are combined in an effective FE.

To gain further insight in decay dynamics and the absorption mechanism, we now investigate pump power dependencies. As seen in **Figure S14**, both radiative losses and the mode change at

1 ps after the pump arrives: in the case of resonance broadening, γ_{rad} increases, and the mode increases in amplitude and width. The spectral position scales approximately linearly with the pump power, showing mostly linear absorption causing the refractive index modulation, while the continuity in γ_{rad} furthermore demonstrates continuous tunability.

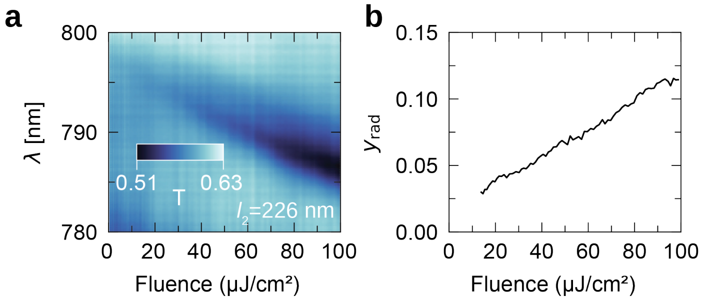

Figure S14: Power dependence at $t = 1$ ps. (a) Pump-fluence dependent transmittance spectrum for $l_2 = 226$ nm (case of resonance creation), showing a gradual increase in resonance amplitude with increasing pump fluence. (b) Corresponding γ_{rad} values derived from fits to the data in (a), demonstrating a continuous increase in γ_{rad} with fluence, reaching up to 0.11 THz.

Decay times of the spectral shift and the radiative loss modulation are shown in **Figure S15a,b**. The latter is generally shorter than the decay time of the spectral shift because the radiative loss depends mostly quadratically on the asymmetry. Furthermore, because of Auger recombination at high carrier densities, the excited populations in the two rods decay at different rates, causing their asymmetry to decay faster than the overall refractive index modulation. However, due to the minor decrease in decay times of the spectral shift at the highest intensities (< 20 %), Auger processes only play a minor role in the observed dynamics.

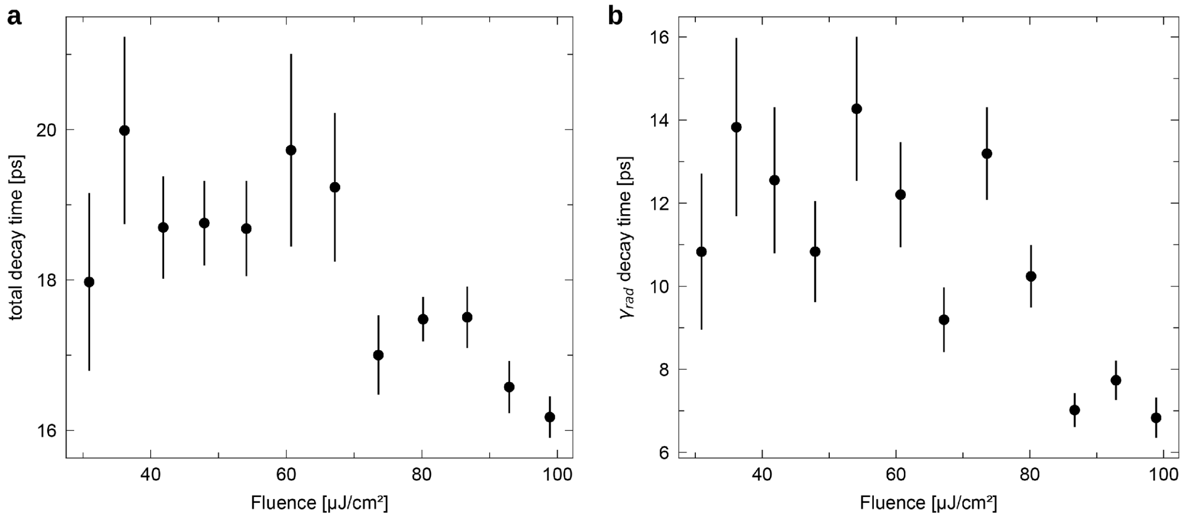

Figure S15: Resonance decay times. (a) 1/e decay time of the pump-induced spectral shift in dependence of the incident pump fluence for the broadening case. Decreasing decay times at high fluences suggest an increasing influence of Auger recombination. (b) 1/e decay time of the fitted radiative loss of the resonance.

Combining these observations allows us to model the decay of the refractive index modulation in good approximation with a single exponential reflecting mostly surface recombination, at a time constant $\tau = 20$ ps for low excitation fluences.

Supplementary Note 8: Estimation of pump-induced refractive index changes

To estimate how both the real (n) and imaginary (k) parts of the refractive index change under laser pumping, we reproduced the mode shift and broadening observed in **Figures 4b** and **5a,b** through numerical simulations. Based on **Figure 3c**, the absorbance, and thus the changes in n and k, is approximately 3.5 times higher in Rod 1 than in Rod 2 once pumped, as illustrated in **Figure S16a**.

Step 1: Estimating Δn: We first determine the change in the real part of the refractive index (Δn) from the shift of the resonance wavelength. In the simulations, silicon is assigned a baseline $n = 3.67$ and $k = 0.006$ (following Schinke et al.[8] around 790-800 nm). To precisely match the unpumped experimental resonance wavelength (i.e., $t = -1$ ps) with the simulated resonance wavelength, we slightly reduce the in-plane dimensions of the unit cell by 2.3%. **Figure S16b** shows the comparison between the experimental (dashed gray curve) and simulated (solid gray curve) spectra. Next, we tune Δn_{rod1} (and hence $\Delta n_{\mathrm{rod1}} \approx 3.5\, \Delta n_{\mathrm{rod2}}$) until the pumped resonance wavelength at $t = 1$ ps spectrally matches with the experimental results (blue curves). This causes a total shift of about 10 nm, which we reproduce numerically using $\Delta n_{\mathrm{rod1}} = -0.18$. Sweeping Δn_{rod1} from 0 to −0.18 continuously shifts the resonance and simultaneously increases its amplitude, consistent with rising radiative loss γ_{rad} as shown in **Figure S16c**.

Step 2: Estimating Δk: With $\Delta n_{\mathrm{rod1}} = -0.18$ ($\Delta n_{\mathrm{rod2}} = -0.05$, hence $\Delta n = -0.13$) fixed, we estimate the accompanying change in the imaginary part of the refractive index. Because the simulation only accounts for intrinsic material loss, we first determine an effective $k = k_{\mathrm{est}}$ that reproduces the pre-pump non-radiative loss γ_{int} measured experimentally (0.94 at $t = -1$ ps, **Figure S12**). Sweeping the same k in both rods (**Figure S17a,b,c**) shows γ_{int} varying linearly with k while γ_{rad} is unaffected; a linear fit yields $k_{\mathrm{est}} = 0.022$ (black line), up from the literature Si value k = 0.006 (Schinke et al.[8]). To find the pump-induced change we assume linear absorption and the same 3.5:1 imbalance as for Δn, i.e. $\Delta k_{\mathrm{rod1}} = 3.5 \Delta k_{\mathrm{rod2}}$ and $\Delta k = \Delta k_{\mathrm{rod1}} - \Delta k_{\mathrm{rod2}}$ (**Figure S17a**). Using the post-pump value $\gamma_{\mathrm{int}} = 1.04$ at $t = 1$ ps (**Figure 3e**), the linear fits in **Figure S17d,e** give $\Delta k_{\mathrm{rod1}} \approx 0.004$ (blue line, $\Delta k \approx 0.003$). Thus, the pump increases k by only a few 10^{-3}, consistent with our observation that the index contrast is dominated by Δn, while Δk contributes only a minor fraction of the total modulation.

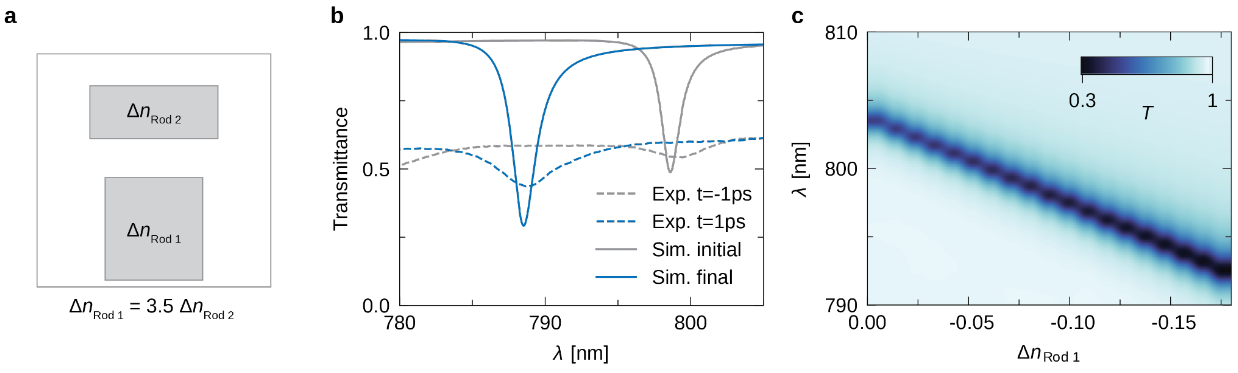

Figure S16: Estimating Δn. (a) From **Figure 3c**, pumping leads to a 3.5 times higher absorbance in Rod 1 than in Rod 2, leading to $\Delta n_{\mathrm{rod1}} \approx 3.5\,\Delta n_{\mathrm{rod2}}$. **(b)** Experimental spectra at $t = -1$ ps and $t = 1$ ps (dashed gray and blue, respectively) compared to the simulations (solid gray and blue). The in-plane dimensions of the simulated structure are reduced by 2.3% to align spectrally with the unpumped experimental mode. Next, Δn_{rod1} is swept from 0 to −0.18 to match with the pumped resonance wavelength. **(c)** Full Δn_{rod1} sweep from 0 to −0.18, showing a continuous 10 nm spectral shift and an amplitude increase matching the experiment.

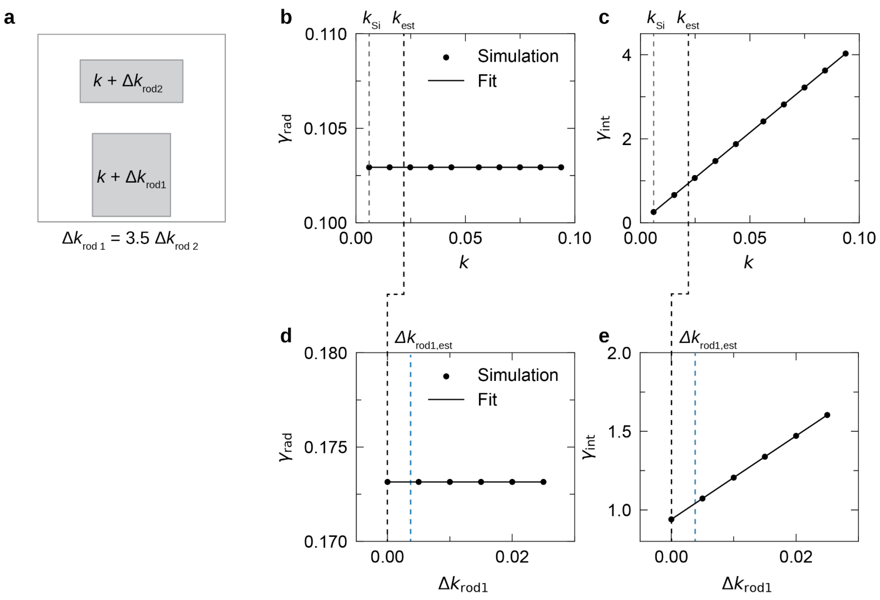

Figure S17: Estimating Δk from pump–probe data. (a) Schematic: besides an initial k value in both rods the pump generates additional absorption with a 3.5:1 imbalance, so $\Delta k_{\mathrm{rod1}} \approx 3.5\,\Delta k_{\mathrm{rod2}}$. (b,c) Pre-pump calibration. Sweeping the intrinsic index k from the literature value 0.006 (grey line) to 0.10 leaves γ_{rad} unchanged (b) but increases γ_{int} linearly (c), matching the experimental $\gamma_{\mathrm{int}} = 0.94$ at $t = -1$ ps (**Figure 3e**) gives $k_{\mathrm{est}} = 0.022$ (black line). (d,e) Post-pump estimate. With k fixed at 0.022, Δk_{rod1} is swept from 0 to 0.025. Again γ_{rad} is unaffected (d), while γ_{int} rises linearly (e); the experimental $\gamma_{\mathrm{int}} = 1.04$ at t = 1 ps (**Figure 3e**) is met for $\Delta k_{\mathrm{rod1}} \approx 0.004$ (blue line), corresponding to $\Delta k \approx 0.003$.

Supplementary Note 9: Potential applications for radiative loss based active photonics

The possible applications for γ_{rad} tuning are diverse. Below, we specifically name six possible directions where γ_{rad} tuning can provide improved performance and new functionalities compared with conventional approaches based on tuning ω_0 or γ_{int}.

1. On/Off Switchable Filters: Conventional approaches to resonance tuning, i.e., shifting ω_0 or increasing γ_{int}, cannot fully switch a resonance on or off. ω_0 tuning moves the mode away from its original spectral position to reduce undesired reflection at that specific wavelength, but it cannot create a completely transparent metasurface system because a highly reflective resonant mode is always present. γ_{int} tuning increases intrinsic losses and thus quenches the reflectance amplitude of the resonance, but still leaves some broadened residual mode, as the structure remains radiatively coupled, just with smaller amplitude and broader linewidth. Furthermore, it typically leads to an overall lower transmission. In contrast, γ_{rad} tuning can entirely decouple the mode from the far field using a system that transitions from truly "resonance-free" ($\gamma_{\text{rad}} = 0$) to fully "resonant" ($\gamma_{\text{rad}} > 0$). The result is an on/off switchable filter that, in its "off" state, is transparent, minimizing spectral crosstalk and absorption. This capability is important for advanced active optical filtering in photonic circuits.

2. On-Demand Sensors: In sensing applications, the ability to activate or deactivate a resonance at will can help to keep an optical system transparent and only switch on resonant modes temporally for sensing if needed. ω_0 tuning leads to resonant modes that are always present, and thus, for some wavelengths, the system is highly reflective. γ_{int} tuning introduces losses into the system and reduces optical transparency for resonant and off-resonant wavelengths. γ_{rad}-driven sensors can remain fully transparent in "off" mode, transmitting the entire spectral band, and then selectively switch "on" the resonance only when particular analytes need to be detected. This is especially advantageous in fiber-based optical systems, where the sensor element remains transparent during normal operation and can be optically activated to create a high-Q response for refractive index sensing.

3. Polaritonic Critical Coupling: Light-matter coupling strength for polariton formation depends on the balance between photonic and material losses. Typically, the photonic mode's total loss $\gamma_{\text{photon}} = \gamma_{\text{rad}} + \gamma_{\text{int}}$ should match or be similar to the material excitation's linewidth γ_{mat} to reach so-called polaritonic critical coupling.[9] Thus, there is a need to actively tune the photonic loss rates to probe and optimize this ratio. ω_0 tuning merely shifts the mode's center frequency and cannot reduce or increase the total loss channel. γ_{int} tuning, meaning increasing intrinsic cavity losses, quenches near-field enhancement and can outweigh the material excitation, effectively preventing polariton formation. The γ_{rad} tuning advantage lies in keeping γ_{int} low and only altering the radiative part of the photonic loss so that it matches the material's loss.

4. Emission Linewidth Control and Tunable Lasing: Many photonic devices rely on controlling the emission properties, such as linewidth and coherence, of an integrated gain medium. In many applications, there is a need to actively control these properties. ω_0 tuning shifts the resonant frequency of the photonic mode, but does not fundamentally alter its damping, so the emission linewidth cannot be changed. γ_{int} tuning induces additional intrinsic losses and reduces the overall emission strength, as further nonradiative decay channels are introduced. γ_{rad} tuning preserves the low nonradiative intrinsic losses of the photonic mode while allowing the radiative decay rate and thus the linewidth to be freely adjusted. For lasing applications, the threshold condition depends on the total cavity losses, and if γ_{rad} is too low, it can be difficult to couple pump light into the cavity or out-couple coherent photons, while if γ_{rad} is too high, the threshold power increases. Dynamically controlling γ_{rad} allows optimization of the cavity's Q-factor, enabling broad- and narrow-line lasing modes with adjustable thresholds.

5. Ultrafast Pulse Modulation and Time-Crystal Metasurfaces:

In the spectral vicinity of the quasi-BIC, significant dispersion is induced on propagating light. γ_{rad} based resonance tuning therefore leads to far-reaching control over key material properties on ultrafast timescales, which is a requisite for experimentally achieving time-crystal metasurfaces. Furthermore, it can be used for novel compact pulse-compression systems: By modifying the dispersion on the same timescale as the pulse, a temporally varying dispersion is induced, meaning the front of the pulse propagates at a different speed than its back. Therefore, this scheme controls pulse lengths without splitting them into their spectral components, potentially allowing on-chip solutions.

Compared with γ_{int} tuning, this approach offers two main advantages: First, tuning γ_{rad} enables precise control over how long light stays in the cavity or when it leaks; γ_{int}-based tuning will only destroy the mode by increasing losses, which is unwanted in these applications. Furthermore, the measured metasurface allows for sub-picosecond increases and decreases in γ_{rad} depending on the exact geometry, hence leading to positive or negative tunable delays. For γ_{int}, on the other hand, ultrafast decreases remain elusive.

6. Nonvolatile Encoding of Metasurfaces: Phase change material-based encoding in metasurfaces can be used to store data or to adjust resonance parameters post-processing. Challenges with ω_0 or γ_{int} tuning arise, because using phase-change materials like GST or Sb_2Se_3 for conventional tuning either shifts the resonance or introduces additional absorption, never fully toggling the mode from nonresonant (transparent) to resonant. γ_{rad} tuning, on the other hand, allows straightforward encoding. Starting with a metasurface in its dark state and using a localized optical pulse to partially change the phase in certain unit cells, resonances can be coupled to the farfield in a controlled manner. A subsequent erase step, for example, uniform heating, resets the material to its transparent state. Because γ_{rad} tuning can achieve a genuine "off" resonance, this

leads to reconfigurable devices that stay transparent until selectively written, enabling optical storage, adjustable patterning of wavefronts, or post-processing optimization.

Supplementary Note 10: Influence of Angled Excitation and Collection

To evaluate the effect of angled illumination and collection on the measured data, we analyze the configuration of our confocal setup in detail. Both excitation and collection use 0.25 NA objectives, whose theoretical maximum half-angle is 14.5°. However, for the reasons discussed below, the practical angular spread is significantly smaller. First, although the objective pupil diameter is ∼11 mm, our beam diameter is only ∼5 mm, reducing the maximum incidence angle to 6.7°. Second, the pump and probe beams are deliberately defocused, resulting in large spot sizes on the sample: 49.5 µm for the pump (**Figure S18a**) and 18.8 µm for the probe (**Figure S18b**). Since not all rays within these spots contain the full ±6.7° angular spread, the center of each spot is nearly collimated, while higher angles appear only at the edges. In contrast, the 0.25 NA objective used for collection is in focus, so the sample region from which we collect light is only ∼2 µm in diameter (**Figure S18c**). Overlaying this small collection region with the much larger defocused spots reveals that mostly collimated, or nearly collimated, rays are collected. We can estimate the angular spread in this central collection region using geometric optics and a small-angle approximation. As the angular range across the full 18.8 µm probe spot goes from ±6.7°, the local angle at a radius r is approximately $\theta(r) = \theta_{\mathrm{exc,max}}/r_{\mathrm{exc}} r$ where $r_{\mathrm{exc}} \approx 9.4$ µm is the spot radius and $\theta_{\mathrm{exc,max}} \approx 6.7°$. Evaluating at $r = 1$ µm (the radius of the collection region), the maximal local angle becomes $\theta(1\mu\mathrm{m}) \approx 0.71°$. Simulations show that for both TE- and TM-polarized light, this leads to minor angular shifts of <0.1 and 0.6 nm, respectively. As the studied Q-factors are all substantially below 500 (FWHM around 1.5 nm), these angular spreads have only a minor effect on mode broadening compared to other experimental loss factors like scattering on rough surfaces or varying resonator sizes.

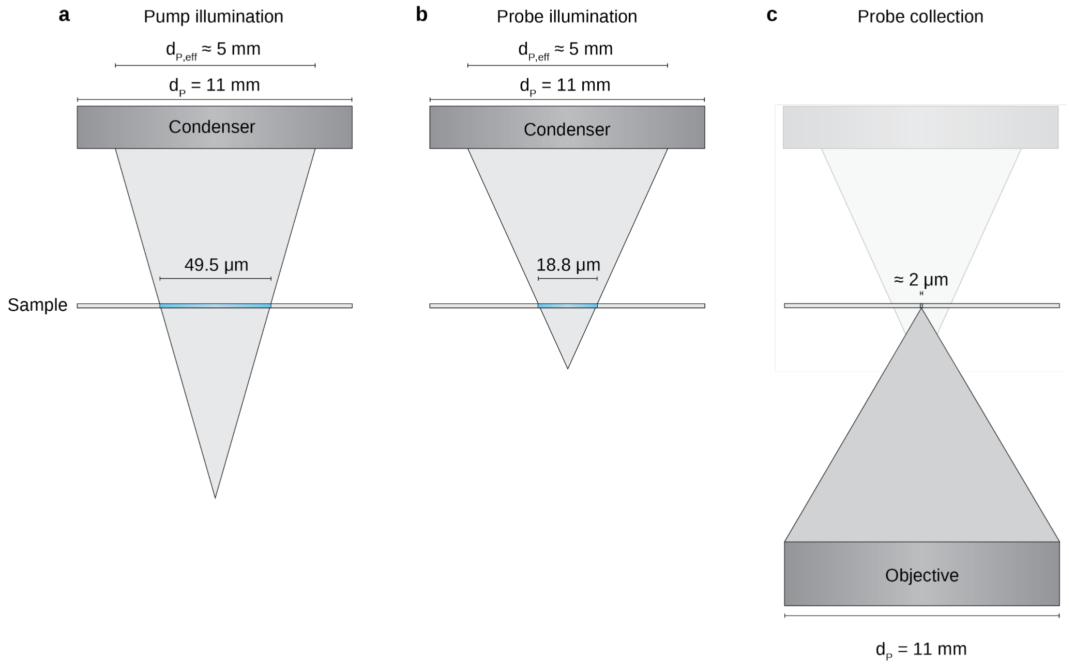

Figure S18: Illumination and collection scheme. Both objectives in the confocal setup have a 0.25 NA with an 11 mm pupil. Since the laser beam diameters are only around ∼5 mm, the effective pupil diameter is smaller, limiting the maximum incidence angle to 6.7°. (a) The pump excitation scheme shows a highly defocused beam, giving a

~49.5 μm full-width at half-maximum (FWHM) spot on the sample. (b) The probe beam is similarly defocused, yielding an ~18.8 μm spot. Both beams pass through the same objective, but their degrees of defocus are independently adjusted with additional lenses. (c) The collection objective is in focus, with an estimated ~2 μm collection area. Although the objective can collect a broader range of angles, a confocal aperture in front of the spectrometer further restricts the light cone.

References

1. Fan, S., Suh, W. & Joannopoulos, J. D. Temporal coupled-mode theory for the Fano resonance in optical resonators. *J. Opt. Soc. Am. A* **20**, 569 (2003).
2. Gorkunov, M. V., Antonov, A. A., Mamonova, A. V., Muljarov, E. A. & Kivshar, Y. Substrate-Induced Maximum Optical Chirality of Planar Dielectric Structures. *Adv. Opt. Mater.* **13**, 2402133 (2025).
3. Koshelev, K., Lepeshov, S., Liu, M., Bogdanov, A. & Kivshar, Y. Asymmetric Metasurfaces with High- Resonances Governed by Bound States in the Continuum. *Phys. Rev. Lett.* **121**, 193903 (2018).
4. Gurvitz, E. A. *et al*. The high-order toroidal moments and anapole states in all-dielectric photonics. *Laser & Photonics Reviews* **13**, 5 (2019).
5. Soref, R. & Bennett, B. Electrooptical effects in silicon. *IEEE J. Quantum Electron.* **23**, 123-129 (1987)
6. Liu, Y.-H. & Cheng, C.-W. The Experimental and Modeling Study of Femtosecond Laser-Ablated Silicon Surface. *J. Manuf. Mater. Process.* **7**, 68 (2023).
7. Goldman, J. R. & Prybyla, J. A. Ultrafast dynamics of laser-excited electron distributions in silicon. *Phys. Rev. Lett.* **72**, 1364 (1994).
8. Schinke, C. *et al.* Uncertainty analysis for the coefficient of band-to-band absorption of crystalline silicon. *AIP Adv.* **5**, 067168 (2015).
9. Weber, T. *et al.* Intrinsic strong light-matter coupling with self-hybridized bound states in the continuum in van der Waals metasurfaces. *Nat. Mater.* **22**, 970–976 (2023).
